# Effectiveness of video-assisted teaching on prevention of constipation among postpartum women admitted in postnatal ward at a tertiary care hospital: a randomised controlled trial

**DOI:** 10.1136/bmjnph-2022-000605

**Published:** 2024-03-14

**Authors:** Tamil Selvi C, Kumari M J, Vahitha S, Kubera N S

**Affiliations:** 1 College of Nursing, Jawaharlal Institute of Postgraduate Medical Education and Research (JIPMER), Puducherry, India; 2 College of Nursing, All India Institute of Medical Sciences, Raipur, India; 3 Department of Obstetrics and Gynaecology, Jawaharlal Institute of Postgraduate Medical Education and Research (JIPMER), Puducherry, India

**Keywords:** Dietary patterns, Nutritional treatment, Nutrition assessment

## Abstract

**Background:**

The postpartum period is a vital phase for a mother as she undergoes a role transition in her life, in addition to physiological changes. Among all discomforts experienced during this period, constipation is more common and it can cause lifelong complications such as haemorrhoids, rectal prolapse and anal fissures if left untreated. Adequate care, education and compliance with proper postpartum diet and exercise can prevent it.

**Aim and objective:**

This study intended to assess the effect of video-assisted teaching in preventing constipation among postpartum women in comparison with routine care.

**Settings and design:**

Antenatal outpatient department and postnatal ward. Experimental research design—randomised controlled trial.

**Methods and material:**

Totally, 160 antenatal women in the III trimester were selected by convenience sampling and randomised into study and control groups. Data were collected using a semistructured questionnaire. Postpartum women in the study group received video-assisted teaching regarding the postnatal diet and exercise for the prevention of constipation developed by the researcher with reference from books, journals, Indian council of medical research Recommended dietary allowances table and expert opinion. Postpartum women in the control group received routine care as a pamphlet regarding the care of women after delivery which was routinely given to all mothers along with the discharge slip. Constipation Assessment Scale was used to assess the presence of constipation at the end of second week of post partum.

**Statistical analysis used:**

Descriptive and inferential statistics were used.

**Results:**

Data showed 27% of postpartum women in control group had constipation comparing to only 6.1% of the women in the study group (p<0.05). There was a significant association between consumption of fruits, green leafy vegetables, increased fluid intake, regular walking and the status of constipation (p<0.001).

**Conclusions:**

Video-assisted teaching was effective in preventing constipation among postpartum women.

**WHAT IS ALREADY KNOWN ON THIS TOPIC**

- Constipation is one of the most common discomfort during puerperium.
- Puerperium is associated with traditional food taboos.
- Dietary fibre and lifestyle modifications can help in preventing constipation.

**WHAT THIS STUDY ADDS**

- Women who received video-assisted teaching had increased consumption of fruits, vegetables and oral liquids and had decreased occurrence of constipation among them.
- Information given using multimedia education such as video-assisted teaching can significantly impact the assimilation of those messages and adapting in practice.

**HOW THIS STUDY MIGHT AFFECT RESEARCH, PRACTICE OR POLICY.**

- Such education videos can be used to educate postnatal women in hospitals as well as in community set-up.
- Further community-based research may reveal the local cultural influences on beliefs and practices of postnatal women based on which interventions can be tailored to their needs.

## Introduction

Maternal health comprises women’s health during pregnancy, childbirth and post partum. Every stage should be a positive experience, allowing women and their babies to reach their full potential for health and well-being.^1^ The postpartum period, lasting from the birth of a baby until 6 weeks after delivery, is a critical time for a mother^2^ as she adjusts to hormonal and physical changes, recuperates from delivery, experiences shifting family responsibilities and suffers sleep deprivation, all while caring for and nourishing their newborns.^3^ Women face additional barriers to maintaining a healthy lifestyle during this period.^4^ Additionally, the demands of caring for a newborn can sometimes lead mothers to prioritise their child’s needs over their health.^5^

Many postpartum problems occur during this period varying from postpartum haemorrhage, pregnancy-related hypertension, pulmonary embolism, puerperal sepsis, wound breakdown, breast abscess and urinary/faecal incontinence to minor ailments such as afterpains, perineal discomfort, constipation, headache, backache and sexual problems. Failure to promptly recognise and address these issues can lead to physical and psychological distress, ultimately diminishing the mother’s quality of life.^6^ Effective postnatal care, characterised by early problem identification and appropriate interventions, is essential for facilitating the mother’s complete recovery and returning to her prepregnancy functional state more quickly.^7^

Postpartum constipation, a common issue after childbirth is characterised by symptoms such as abdominal discomfort, excessive straining, hard and difficult-to-pass stool, lumpy stool and a feeling of incomplete evacuation causes include pregnancy hormones, childbirth-related damage to the anal sphincter or pelvic floor muscles, dietary changes, medication use and reduced physical activity postdelivery.^8 9^ Fear of expected pain at the surgical site, ignoring bowel urges and consuming a low-fibre diet may increase the risk of developing postpartum constipation.^10^

Findings from the Gut health survey conducted by Abbott—a healthcare company, among eight cities in India in 2018 (Mumbai, Delhi, Kolkata, Hyderabad, Chennai, Patna, Ahmedabad and Lucknow) suggested that 22% of the Indian adult population experiencing constipation. It revealed pregnancy (25%, one in four pregnant women) as an important cause, and irregular eating habits, consumption of junk food, less water intake, and a sedentary lifestyle have been identified as crucial factors causing constipation.^11^

Constipation is an unpleasant symptom that can hurt the quality of life.^12^ Excessive straining can damage the pudendal nerve and impair pelvic floor muscle functions.^13^ Left untreated, it can worsen and cause lifelong complications such as haemorrhoids, rectal prolapse and anal fissures.^14^ Most of the difficulties and minor discomforts such as constipation after birth can be prevented during the postpartum period by a proactive approach of providing enough care and education, plus adherence to proper diet and postpartum exercises.^15^

A healthy postpartum lifestyle is vital for optimal maternal health. Non-pharmacological interventions such as diet and physical activity are suboptimal in the general population and, more specifically, postpartum women.^16^ Educating and supporting women to adopt a healthy lifestyle involving changing long-term habits, specifically diet, physical exercise and sustaining this change over a long period helps to minimise the problems and improve the quality of life.^17^

The present study objectives were to compare the effect of video-assisted teaching regarding postnatal diet and exercise with routine care in preventing constipation among postpartum women admitted to the postnatal ward and to find the association between sociodemographic, obstetric, personal variables and constipation status among postpartum women.

## Materials and methods

A randomised controlled trial was conducted to assess the effectiveness of video-assisted teaching on the prevention of constipation among postpartum women in a tertiary care hospital, in the antenatal outpatient department and in the postnatal ward. The study samples were allocated to the study and control groups by block randomisation using the computer-generated random sequence number of block size 4. The sample size was 80 in each group. The sample size was estimated based on comparing two independent proportions using the formula N= {Z1−α/2×√p‾×q‾×(1+1∕k)+Z1−β×√p_1_×q_1_+(p2 × q2∕k)}^2^/Δ^2^ concerning a study done by (Liu *et al*).^1^ By expecting a constipation rate in the intervention group of 12% and a 20% difference in constipation rate between the study and control group at a 5% level of significance with a power of 80%, and by expecting attrition of 20%, the present study targeted to recruit at least 80 subjects in each group for the present study. A computer-generated random number sequence employing block randomisation of size 4 generated by faculty in the department of biostatistics who is not a part of the study was used to randomise the patients. The random sequence was concealed before allocation by the technique (serially numbered opaque sealed envelope). After informed consent, the principal investigator allocated the participant to the corresponding arm. Blinding was ensured for the outcome measurements by an independent examiner unaware of group allocation.

**Figure 1:**
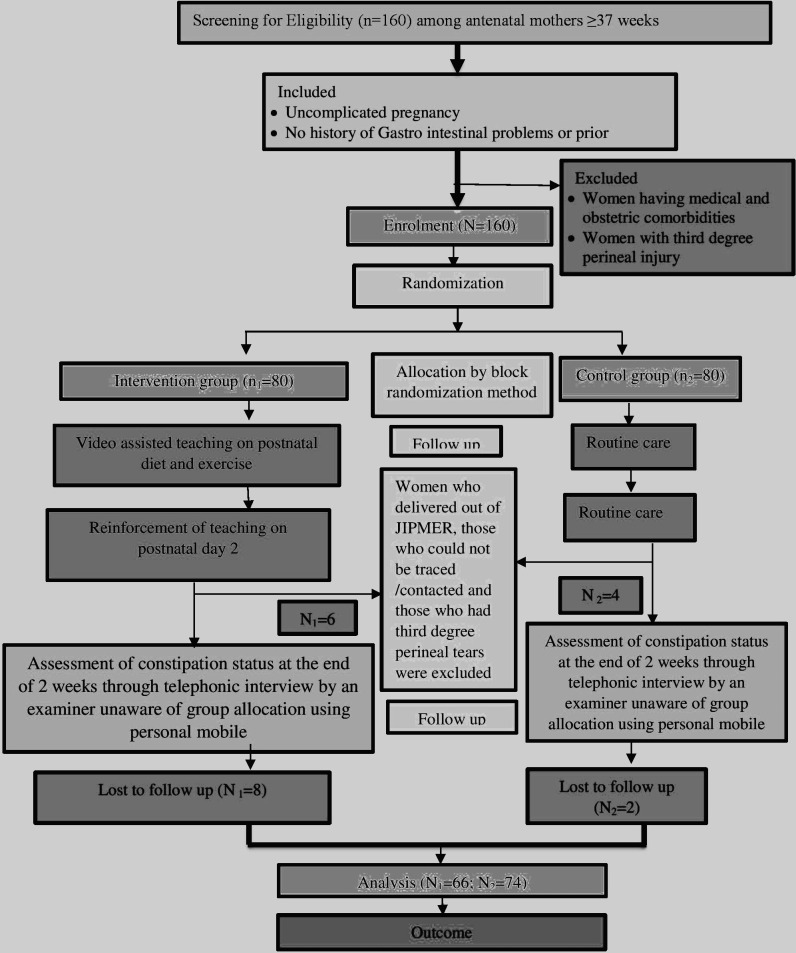
CONSORT diagram. CONSORT, Consolidated Standards of Reporting Trials; JIPMER, Jawaharlal Institute of Postgraduate Medical Education & Research.

The inclusion criteria of the study were uncomplicated pregnancy, no history of gastrointestinal problems or prior surgery and antenatal women ≥37 weeks. Women having medical and obstetrical comorbidities and women with third-degree perineal injury were excluded from the stud (figure 1).

### Development and description of the data collection instrument

A semistructured questionnaire was used for data collection, encompassing various sociodemographic variables such as age, educational background, occupation, income, religion, family structure, residence, dietary habits, as well as obstetrical variables like parity, mode of delivery, iron tablet consumption, past experiences of constipation during prior childbirth and postdelivery practices such as the timing of oral intake. Additionally, personal variables such as fluid intake, consumption of fresh fruits and intake of green leafy vegetables during the postpartum period were also included in the questionnaire, which is collected at the end of 2 weeks of post partum. A constipation assessment scale was used to assess the presence of constipation. The Constipation Assessment Scale for Pregnancy is an adaptation of the Constipation Assessment Scale of McMillan and Williams (by Broussard, 1998), differing essentially in using 5-point rather than a 3-point rating scale for each constipation characteristic. The change was made to produce interval-level data, allowing parametric statistical testing. The validity and reliability of the scale ((r=0.84) reported in JOGNN 27 (3) 297–301 1998) support the ability of the scale to differentiate between the moderate and severe intensity of symptoms.

Constipation assessment scale is a 5-point rating scale consisting of eight statements, each ranging from 0 to 4. The total score is 32, the level of constipation was categorised as 0–8 is none to minimal, 9–17 is mild, 18–24 is moderate and 25–32 is severe constipation.

### Intervention

The study group received video-assisted teaching, where the researcher created an informative video lasting for 20 min based on content derived from books, journals, Indian Council of Medical Research Recommended Dietary Allowances table, and expert opinions covering topics related to constipation, its underlying causes and preventive measures, with a focus on promoting fluid intake, adopting a high-fibre diet and incorporating exercise like walking. These educational sessions were delivered to participants at the time of recruitment and again on the second postnatal day. Patients were told to maintain a record of their dietary pattern and exercise duration in their ANC notebook. Postassessment was done 2 weeks after delivery. Control group received routine care in the form of a pamphlet outlining the care of women after delivery, which was routinely given to all mothers along with the discharge slip.

### Data collection procedure

Sociodemographic and obstetric variables were collected by means of interview method using a questionnaire. Women in the study group received a 20 min video teaching session on postnatal diet and exercise to prevent constipation on enrolment. The researcher checked the labour room and postnatal ward daily to identify the study participants who had delivered. After delivery, women in the study group received another video teaching session on the second day. The control group received routine care, consisting of a postnatal care pamphlet on discharge. The women who delivered outside JIPMER, those who could not be traced/contacted and those who had third-degree perineal tears (n_1_=6; n_2_=4) were dropped from the study. As the educational sessions were reiterated on the second postpartum day, excluding participants who gave birth outside the JIPMER facility. Women who delivered in JIPMER (n_1_=74; n_2_=76) were followed up via mobile phone. Constipation assessment was conducted through telephonic interviews by an independent examiner, blind to group allocation, using the researcher’s mobile. Responses were recorded, and those with constipation received health teaching over the phone. Participants unreachable by phone were excluded since follow-up and post-test data were not available (n_1_=8; n_2_=2). Data from the remaining participants (n_1_=66; n_2_=74) were analysed using ‘R software for statistical computing and graphics V.4.1.2’ downloaded from https://www.r-project.org/. Confidentiality was assured and the data collection spanned over 9 weeks from 3 December 2021 to 2 February 2022.

## Results

Demographic variables of postpartum women in the study and control groups show that most postpartum women in both study group 38 (57.6%) and control group 38 (51.4%) are under the age group of 20–25 years. Regarding education, 41 (62.1%) of the study group are graduate/postgraduate, while 44 (59.5%) of the control group completed school education. In study group, 63 (95.5%) and 71 (95.9%) of control group are homemakers. Family monthly income ranges from Rs. 5000 to Rs. 10 000 for 36 (54.5%) in the study group and 32 (43.2%) in the control group. Furthermore, 48 (72.7%) of the study group and 49 (66.2%) of the control group belong to joint family. The groups are comparable in education, income and family type (p<0.05). The median age with an IQR are 24.7 (IQR 22–27) for the study group and 25.5 (IQR 23–28.75) for the control group. Mann-Whitney U test indicates no significant age difference between the groups (p>0.05).

Obstetric variables indicate that most postpartum women in both study group 47 (71.2%) and control group 46 (62.2%) are primiparous, with a majority in both groups (44 (66.7%) in study, 49 (66.2%) in control) underwent normal vaginal delivery. Additionally, the majority in both groups, that is, 48 (72.7%) in the study vs 56 (75.7%) in the control had oral intake within 2 hours after delivery. The groups were comparable in terms of mode of delivery (p<0.05).

Regarding personal variables, a higher proportion of women in the study group 58 (87.9%) compared with 35 (47.3%) in control group consume fruits daily. Additionally, in the study group, 29 (43.9%) of women consume green leafy vegetables 2–3 times per week while in the control group, the majority 60 (81.1%) do not consume green leafy vegetables at all. The groups are comparable in terms of daily activities, daily fruit intake and intake of green leafy vegetables (p<0.001).

Online supplemental table summarises constipation-related signs and symptoms among postpartum women in study and control groups in the postassessment. Table 1 illustrates the level of constipation in these groups in postassessment. Table 2 compares the constipation status between the study and control groups post assessment. Results indicate significant association between education, family income, daily liquid intake, daily activities, daily fruits intake and intake of green leafy vegetables with constipation status. However, there is no significant association between obstetric variables and constipation status.

10.1136/bmjnph-2022-000605.supp1 Supplementary data

**Table 1:** Level of constipation among postpartum women in study and control groups in postassessment

Level of constipation	Study group (n_1_=66)		Control group (n_2_=74)	
	N	%	N	%
None	62	93.9	54	73
Mild	4	6.1	20	27
Moderate	0	0	0	0
Severe	0	0	0	0

**Table 2:** Comparison of status of constipation among postpartum women in study and control group in postassessment

Status of constipation	Study group (n_1_=66)		Control group (n_2_=74)		χ^2^ value	P value
	N	%	N	%		
Yes	4	(6.1)	20	(27)	**9.3709**	**0.002205**
No	62	(93.9)	54	(73)		

Stastically signicant P-values are indicated in bold.

N = frequency; n_1_ = study group; n_2_ = control group; χ^2^ = chi-square.

## Discussion

The primary objective of this study is to compare the effect of video-assisted teaching regarding postnatal diet and exercise with routine care on preventing constipation among postpartum women. The findings indicate that 6.1% of postpartum women in the study group experienced constipation, whereas 27% of women in the control group had constipation. The difference between the groups was statistically significant with χ^2^=9.37 and p=0.0022.

The study revealed that women who received video-assisted teaching showed a notable increase in their intake of fruits, vegetables and oral liquids, leading to a decrease in constipation among them. It highlights the effectiveness of multimedia education, such as video-assisted teaching on diet and exercise, in preventing postpartum constipation compared with the conventional method of distributing printed pamphlets.

A similar result was detected in a study by Quan *et al*.^15^ They conducted a randomised controlled trial to study the influences of multimedia health education on constipation and negative emotions in puerperal women of China. The results revealed a significant difference in constipation and negative emotions between groups 3, 10, 20 and 30 days after childbirth (p<0.01). Thus, the study concluded that multimedia education could lower the occurrence of constipation among puerperal women.

The second objective of the study is to find the association between sociodemographic, obstetric, and personal variables and constipation status. Sociodemographic variables such as education and income had shown a statistically significant association with constipation status (p<0.05). However, obstetric variables, including parity, mode of delivery, time of initiation of oral intake and consumption of iron tablets, had no association with constipation status. Results showed a statistically significant association between personal variables such as daily liquid intake, daily activities, daily fruit intake, intake of green leafy vegetables and constipation status (p<0.001). This underscores the importance of increasing the oral fluids, fruits and green leafy vegetable intake in the daily diet to alleviate discomfort like constipation among postpartum women, thereby enhancing their enjoyment of motherhood.

Subsequent studies, such as a cross-sectional study by Qin *et al* 18 among postnatal women in urban and suburban areas of China to explore the association of postpartum dietary quality and behavioural practices with maternal health, supported these findings. Constipation (23.6%) was one of the problems studied. The study revealed that breast feeding, postpartum exercise, and higher fish intake, fruits, vegetables, and milk were protective factors against such problems.

## Conclusion

The study evaluated the effectiveness of video-assisted teaching in preventing constipation among postpartum women when compared with routine care. It manifested that multimedia tools, such as video-assisted teaching, have a significant influence on the understanding and implementation health-related messages among postpartum women. Consequently, the study concluded that video-assisted teaching is the most effective educational tool for preventing constipation among postpartum women and encouraging adopting healthy lifestyle.

### Recommendation

Constipation, if left untreated, can result in complications such as haemorrhoids, anal fissures and rectal prolapse, placing additional burden on women who are responsible for both their own well-being and that of their newborns, ultimately impacting their quality of life negatively. Non-pharmacological interventions, including dietary modifications such as increasing fluid intake and fiber-rich foods and early ambulation, are recommended to prevent this problem. Using appealing audio-visual aids for education by healthcare providers would likely be more effective in promoting women’s health in this regard.

### Limitations of the study

- Though the constipation assessment tool was used to assess constipation, it is a subjective symptom, and postnatal women’s individual perceptions may influence their scores for each aspect of the tool.
- Video-assisted teaching was solely provided to postnatal women, excluding their family members, whereas their food habits and health behaviours are largely influenced by their family members.

### Relevance of the study

According to the study findings, video-assisted teaching proves more effective in promoting healthy dietary practices and preventing constipation among postpartum women compared with the traditional practice of issuing printed pamphlets.

## Data Availability

Data are available on reasonable request.
