# Supplementary material for: Effectiveness of video-assisted teaching on prevention of constipation among postpartum women admitted in postnatal ward at a tertiary care hospital: a randomised controlled trial
Source: BMJ Nutr Prev Health. 2024 Mar 14;7(1):128–32. doi: 10.1136/bmjnph-2022-000605 (PMC11221277; doi:10.1136/bmjnph-2022-000605)
Supplement: Supplementary data [file bmjnph-2022-000605supp001.pdf]

Supplementary table: Constipation related signs and symptoms among postpartum women in study and control groups in post assessment
N=66

Signs and symptoms of constipation	None n (%)		Mild n (%)		Moderate n (%)		Severe n (%)		Very severe n (%)	
	Study group	Control group	Study group	Control group	Study group	Control group	Study group	Control group	Study group	Control group
Abdominal distension or bloating	66 (100)	74 (100)	0 (0)	0 (0)	0 (0)	0 (0)	0 (0)	0 (0)	0 (0)	0 (0)
Change in amount of gas passed rectally	65 (98.5)	64 (86.5)	1 (1.5)	10(13.5)	0 (0)	0 (0)	0 (0)	0 (0)	0 (0)	0 (0)
Less frequent bowel movements	62 (93.9)	54 (73)	0 (0)	0 (0)	4 (6.1)	19 (25.7)	0 (0)	1 (1.3)	0 (0)	0 (0)
Oozing liquid stool	66 (100)	74 (100)	0 (0)	0 (0)	0 (0)	0 (0)	0 (0)	0 (0)	0 (0)	0 (0)
Rectal fullness or pressure	62 (94)	54 (73)	3 (4.5)	8 (10.8)	1 (1.5)	12 (16.2)	0 (0)	0 (0)	0 (0)	0 (0)
Rectal pain with bowel movement	62 (93.9)	54 (73)	0 (0)	0 (0)	0 (0)	1 (1.3)	4 (6.1)	19 (25.7)	0 (0)	0 (0)
Small volume of stool	62 (94)	54 (73)	3 (4.5)	12 (16.2)	1 (1.5)	8 (10.8)	0 (0)	0 (0)	0 (0)	0 (0)
Unable to pass stool	62 (93.9)	54 (73)	0 (0)	0 (0)	4 (6.1)	17 (23)	0 (0)	3 (4)	0 (0)	0 (0)
